# Brain Levels of Catalase Remain Constant through Strain, Developmental, and Chronic Alcohol Challenges

**DOI:** 10.1155/2012/572939

**Published:** 2012-08-05

**Authors:** Dennis E. Rhoads, Cherly Contreras, Salma Fathalla

**Affiliations:** Biology Department, Monmouth University, West Long Branch, NJ 07764, USA

## Abstract

Catalase (EC 1.11.1.6) oxidizes ethanol to acetaldehyde within the brain and variations in catalase activity may underlie some consequences of ethanol consumption. The goals of this study were to measure catalase activity in subcellular fractions from rat brain and to compare the levels of this enzyme in several important settings. In the first series of studies, levels of catalase were compared between juvenile and adult rats and between the Long-Evans (LE) and Sprague-Dawley (SD) strains. Levels of catalase appear to have achieved the adult level by the preadolescent period defined by postnatal age (P, days) P25–P28, and there were no differences between strains at the developmental stages tested. Thus, variation in catalase activity is unlikely to be responsible for differences in how adolescent and adult rats respond to ethanol. In the second series of studies, periadolescent and adult rats were administered ethanol chronically through an ethanol-containing liquid diet. Diet consumption and blood ethanol concentrations were significantly higher for periadolescent rats. Catalase activities remained unchanged following ethanol consumption, with no significant differences within or between strains. Thus, the brain showed no apparent adaptive changes in levels of catalase, even when faced with the high levels of ethanol consumption characteristic of periadolescent rats.

## 1. Introduction

Ethanol consumption and subsequent oxidation lead to acetaldehyde production both peripherally and within the central nervous system [1]. Acetaldehyde appears to be a psychoactive substance with, for example, reinforcing properties that may be greater than that of ethanol itself [1–4]. Catalase (EC 1.11.1.6) is responsible for the majority of acetaldehyde production in the brain [5, 6]. Moreover, modulation of catalase levels can alter behavioral responses to ethanol, presumably by controlling levels of acetaldehyde [7–10] and/or by influencing the rate of ethanol elimination within the brain [11]. We are interested in whether natural variation in catalase between developmental stages might account for differences in behavioral responses to intoxicating doses of alcohol. Compared to adults, adolescent rats are less sensitive to loss of motor coordination, less sensitive to the sedative and anxiolytic effects of ethanol, and more sensitive to effects on memory [12–14]. These differences have been interpreted in light of ethanol itself, but relative levels of acetaldehyde production could be a complicating factor in this interpretation if, for example, catalase varied as a function of the developmental stage.

 Adolescents may also differ in response to chronic ethanol. Using an ethanol-containing liquid diet to administer alcohol chronically, adolescent Long-Evans (LE) rats were shown to consume high levels of alcohol and to develop severe withdrawal symptoms consistent with alcohol dependency [15]. Ethanol consumption and severity of the resulting alcohol withdrawal syndrome both decreased as the rats aged through and beyond the periadolescent period. Liver alcohol metabolism and ethanol elimination rates were as high or higher in adolescent rats compared to adults [15]. The present study focused on brain catalase, another potentially important pharmacokinetic factor in this model. In addition, adolescents of the Sprague-Dawley (SD) strain consumed comparable levels of ethanol to the LE adolescents but had lower withdrawal severity [15, 16]. Thus, a side-by-side comparison between the two strains was conducted before and after chronic ethanol consumption.

## 2. Materials and Methods

### 2.1. Animals and Ethanol Feeding

Male LE and SD rats were obtained from Charles River Laboratories (Raleigh, NC, USA) and used after at least 3 days of acclimation to our animal facility. Rats were maintained in a controlled temperature and humidity environment with a light cycle from 0700 to 1900. In studies of chronic ethanol treatment, rats were housed individually and fed for 3 weeks with a preformulated liquid diet [17] (LD'82 Liquidiets, Bioserv Inc., Frenchtown, NJ, USA) as described previously [15]. Rats had unlimited access to the ethanol-containing diet, and the amount of diet consumed was recorded daily for each rat. Age-matched controls were pair-fed an ethanol-free liquid diet or given free access to rat chow and water. Previous work with different periods of ethanol consumption showed that 3 weeks were sufficient to result in a high frequency of severe withdrawal symptoms in LE rats beginning alcohol consumption at postnatal age (P, days) P25 and to expose differences between P35 LE and SD rats [15]. Therefore, for this side-by-side comparison of strains, we determined levels of catalase in groups of preadolescent juveniles (P25–28), while additional P25–28 groups of each strain began consuming an ethanol-containing diet and continued for 3 weeks into the normal adolescent period [12]. Age-matched controls were available for comparison at the end of this 3-week period. In addition, catalase activities were determined in ethanol-naïve adults of each strain (>P75), and then groups of adults were given the ethanol-containing diet for the same 3-week period. All protocols involving rats were reviewed and approved by the Institutional Animal Care and Use Committee of Monmouth University as prescribed in the Public Health Service Guide for Care and Use of Laboratory Animals.

### 2.2. Determination of Blood Ethanol Concentration (BEC)

As described previously [16], BEC was determined from trunk blood without withdrawing the rats from the alcohol-containing diet. Rats were sampled between 7:30 and 11:30 am, that is, in the first third of the light cycle. A commercial kit (QuantiChrom Ethanol Assay Kit, BioAssay Systems, Hayward, CA, USA) was used to determine levels of blood alcohol.

### 2.3. Fractionation of Rat Brain

Rats were sacrificed by rapid decapitation and brain was removed by dissection on ice. Crude nerve-ending (synaptosomal/mitochondrial) fractions, and post-mitochondrial supernatant fractions were prepared from brain homogenates by differential centrifugation using a modification of the original method of Gray and Whittaker [17] as described previously [16, 18]. Based on the brain fractionation scheme used by Zimatkin and coworkers [5], we assumed that this crude synaptosomal/mitochondrial fraction would contain some portion of the catalase-containing microperoxisomes. In some studies, synaptosomes were enriched by Ficoll density gradient centrifugation [18]. As described previously [16], resuspension and recentrifugation of fractions was used to remove any ethanol that may have been carried over from *in vivo* ethanol administration (*in vitro* withdrawal). Fractions were subdivided into aliquots and stored at −70°C prior to use. Protein concentrations were determined by the Bradford method (Quick Start Bradford Assay Kit, Bio-Rad Laboratories, Hercules, CA, USA) utilizing bovine serum albumin as the protein standard.

### 2.4. Measurement of Catalase Activity

Catalase was measured spectrophotometrically using a coupled assay system available commercially (Edvotek, Bethesda, MD, USA). In brief, the assay included 7 mM H_2_O_2_ as the substrate for catalase and followed loss of H_2_O_2_ over time as the measure of the initial rate of catalase activity (expressed as *μ*mol H_2_O_2_ min^−1^·mg protein^−1^). Preliminary trials indicated that this initial concentration of H_2_O_2_ substrate yielded activity values approximating *V*_max⁡_ and providing an estimate of the amount of enzyme present in the brain fractions. In this assay system, H_2_O_2_ was determined after reaction with KI. Brain fractions were solubilized for 10 minutes on ice with 0.1% sodium deoxycholate prior to use as the source of enzyme. Except for the source of enzyme, assay conditions were the same for all subcellular fractions tested. Initial trials determined that the reduction in [H_2_O_2_] was linear for each fraction over the time range tested.

### 2.5. Data Analysis

Results are expressed as mean ± SEM. Ethanol consumption, BEC, and catalase were each compared among treatment groups by two-factor ANOVA (2 strains × 2 developmental stages with repetition) using ProStat (Poly Software International, Pearl River, NY, USA). Within-strain analysis of ethanol-fed and age-matched control rats was also conducted by two-factor ANOVA (2 treatment groups × 2 developmental stages with repetition). Where necessary, Tukey's post hoc test was used for multiple comparisons. In all cases, significance was set at *P* < 0.05.

## 3. Results

### 3.1. Catalase Activity in Ethanol-Naïve Rats

Catalase was detected in the crude synaptosomal/mitochondrial fractions and used initially to compare ethanol-naïve LE and SD rats at the two developmental stages (Table 1). Two-factor ANOVA indicated that there was no significant main effect of developmental stage (preadolescent versus adult) (*F*(1,36) = 0.555,  *P* = 0.461) and no significant main effect of strain (LE versus SD) (*F*(1,36) = 0.732,  *P* = 0.398). There was also no significant interaction effect between strain and developmental stage (*F*(1,36) = 1.547,  *P* = 0.222).

 It was of interest to determine if catalase was enriched in the nerve-ending particles (synaptosomes) from this fraction. For preadolescent rats (P25–28), preparations averaging 1.39 *μ*moles·min^−1^·mg protein^−1^ in the crude fraction had 3.93 *μ*moles·min^−1^·mg protein^−1^ catalase activity in the enriched synaptosomal preparation. For adult rats, synaptosomal preparations averaged 2.40 *μ*moles·min^−1^·mg protein^−1^ catalase activity compared to 1.18 *μ*moles·min^−1^·mg protein^−1^ in the crude fraction. Thus, activity was increased 2-3-fold when the preparation was enriched for the synaptosomes. However, based on the yield of synaptosomes, catalase activity in the enriched fraction averaged less than 10% of the total activity present in the crude fraction. For this reason, it was decided to continue using the crude fraction for screening the strains and developmental stages after treatment with ethanol.

### 3.2. Chronic Ethanol Consumption

**Figure 1:**
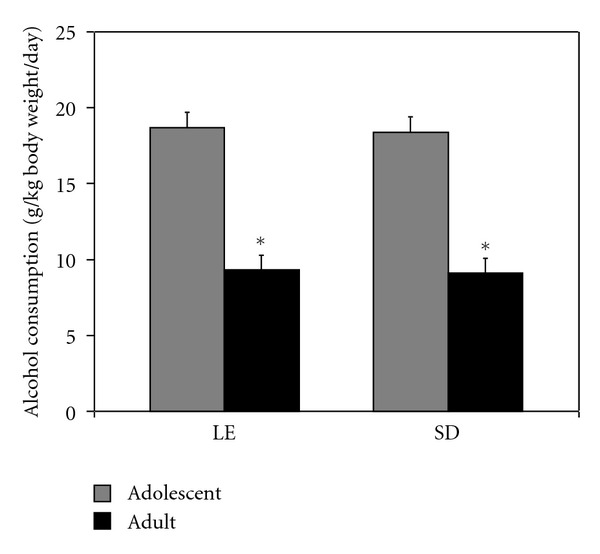
Alcohol consumption was significantly higher for periadolescent rats. Two strains of rats (Long-Evans and Sprague-Dawley) consumed an ethanol-containing liquid diet for three weeks starting either as preadolescents at postnatal age 25–28 days (P25–28) or as adults (P75 or greater). Average daily ethanol consumption (g ethanol day^−1^ kg body weight^−1^) was determined over the course of treatment and presented as mean ± SEM. *For each strain, there was a highly significant difference in alcohol consumption between the periadolescents and adults (*P* < 0.001).

Additional rats of each strain were fed ethanol as part of a liquid diet starting either as preadolescents (P25–28) or adults (at least P75). Average consumption of alcohol over the course of treatment was determined for each of the 2 strains at each of the two developmental stages (Figure 1). Two-factor ANOVA showed there was a significant main effect of developmental stage (*F*(1,28) = 158.460,  *P* < 0.001) with adolescents consuming significantly more alcohol than the adults. The level of consumption for adolescent rats averaged 18.5 g ethanol/day/kg body weight compared to average consumption of 9.2 g ethanol/day/kg body for the adult rats. There was no significant effect of strain (*F*(1,28) = 0.083,  *P* = 0.776) and no interaction effect between strain and developmental stage (*F*(1,28) = 0.007,  *P* = 0.933) on alcohol consumption.

**Figure 2:**
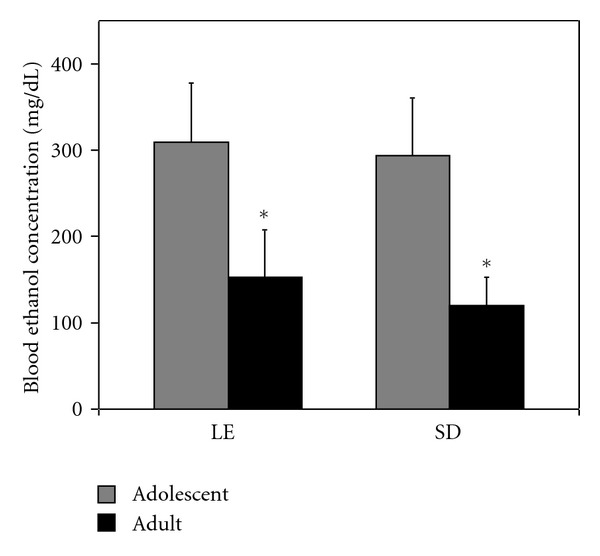
Blood ethanol concentrations were significantly higher for periadolescent rats. Following three weeks of consuming an ethanol-containing liquid diet, Long-Evans and Sprague-Dawley rats which began diet consumption as preadolescents at postnatal age 25–28 days (P25–28) or as adults (P75 or greater) were sacrificed, and blood ethanol concentration (BEC) was determined in trunk blood and presented as mean ± SEM. *For each strain, there was a highly significant difference in BEC between the periadolescent rats and adults (*P* = 0.003).

 Blood ethanol concentrations (BECs) appeared to be consistent with the differences in alcohol consumption between adults and adolescents of each strain (Figure 2). Two-factor ANOVA again yielded a significant main effect of developmental stage (*F*(1,20) = 11.126, *P* = 0.003) with BEC significantly higher for adolescents than adults. There was no significant effect of strain (*F*(1,20) = 0.001, *P* = 0.97) and no interaction effect between strain and developmental stage (*F*(1,20) = 0.090, *P* = 0.768) on BEC.

**Figure 3:**
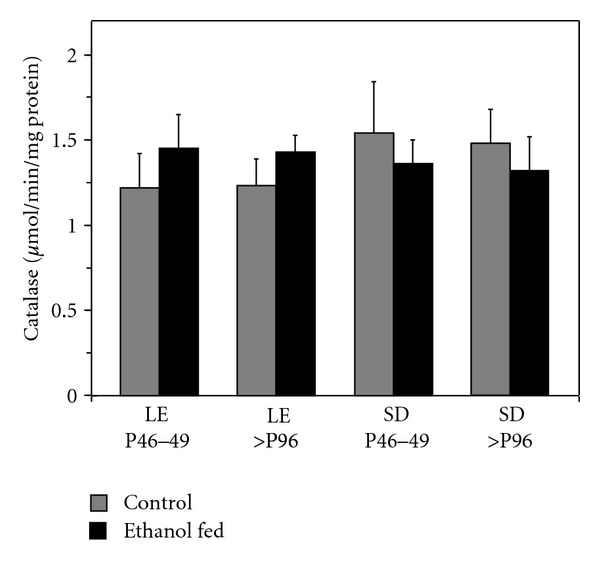
Within strain comparisons show no difference between control and ethanol-fed rats. Age-matched rats of the Long-Evans (LE) and Sprague-Dawley (SD) strains were pair-fed control or ethanol-containing diets for three weeks starting either as: (1) preadolescents at postnatal age 25–28 days (P25–28) ending treatment at P46–49 or as (2) adults (P75 or greater) ending treatment at P96 or greater. Rats were sacrificed at the end of the treatment period, and the brains were dissected for determination of catalase activity in synaptosomal fractions solubilized with 0.1% sodium deoxycholate. The age for each treatment group pair at the time of analysis is shown. Catalase activity is expressed as *μ*mol H_2_O_2_ min^−1^·mg protein^−1^ and presented as mean ± SEM. For each treatment group pair, there was no significant difference in synaptosomal catalase activity.

 Given differences in ethanol consumption and BEC, levels of catalase were determined in ethanol-fed rats where rats of each strain began alcohol consumption either as pre-adolescents or as adults. To assess directly whether ethanol consumption altered catalase within strains, catalase levels in ethanol-fed rats were compared to pair-fed, age-matched controls (Figure 3). For the Long-Evans strain, two-factor ANOVA indicated that there was no significant main effect of treatment group (ethanol-fed versus control) (*F*(1,36) = 3.799, *P* = 0.059) and no significant main effect of developmental stage (periadolescent versus adult) (*F*(1,36) = 0.000, *P* = 0.985). There was also no significant interaction effect between strain and developmental stage (*F*(1,36) = 0.038, *P* = 0.846). Similar results were obtained for within-strain analysis of the Sprague-Dawley rats. Two-factor ANOVA indicated there was no significant main effect of treatment group (ethanol-fed versus control) (*F*(1,18) = 0.580, *P* = 0.456) and no significant main effect of developmental stage (periadolescent versus adult) (*F*(1,18) = 0.049, *P* = 0.827). There was also no significant interaction effect between strain and developmental stage (*F*(1,18) = 0.003, *P* = 0.956). Thus, this period of ethanol administration did not result in significantly altered levels of catalase compared to age-matched control animals. This was true for both the lower levels of alcohol consumption and BEC seen with the adults and the significantly higher levels of consumption and BEC seen with the adolescents. To be sure that the lack of effect of ethanol was not unique to the crude synaptosomal fraction, we spot checked the results for a postmitochondrial fraction. For Long-Evans rats beginning alcohol consumption as preadolescents, the level of catalase in the postmitochondrial supernatant was 1.49 ± 0.22 *μ*moles·min^−1^·mg protein^−1^. For the corresponding age-matched controls, the level of catalase in the post-mitochondrial supernatant was 1.70 ± 0.13 *μ*moles·min^−1^·mg protein^−1^. The difference with ethanol feeding was not significant (*P* > 0.05). Thus, alcohol feeding under the conditions used did not alter catalase activity in either the crude synaptosomal or post-mitochondrial fractions.

## 4. Discussion

In previous studies of rat brain development, catalase levels were reported to be highest immediately after birth and then to decrease to adult levels by P30 [19, 20]. These changes were observed in several different brain regions [19]. In comparing levels between preadolescent rats and young adults (>P75), it appears in our study that the adult catalase level had been reached by P25–28 in LE and SD rats. Although it may be enriched in aminergic neurons [21], catalase is widely distributed and largely associated with microperoxisomes in the postnatal rat brain [22]. Associated with oxidative stress, catalase has been reported for adult Wistar rats to decrease in homogenates of liver, brain, and other tissues following 4 weeks of ethanol administration by oral gavage [23]. Decreases in superoxide dismutase and glutathione peroxidase were also observed. Similarly, modest (~20%) but significant decreases in catalase were observed in adult and aged Wistar rats after 4 weeks consuming an ethanol-containing liquid diet [24]. However, other studies showed no change in liver catalase with adult SD rats after 2 weeks of ethanol exposure through drinking water [25] and with adult Wistar rats after a single dose [26]. For mice, variations in response of liver catalase to ethanol varied by strain from slight induction to decreases [26]. Thus, duration of ethanol exposure and genetic background appear to be important variables in considering whether or not catalase changes as a response to ethanol. It is important to note that the responses of adolescent rats have not been reported previously. Thus, the present study expands on this past work in three important ways.

First, we have shown that catalase can be measured in crude and enriched nerve-ending (synaptosomal) fractions. Different subcellular fractions have been used to measure brain catalase including homogenates [20, 23], mitochondrial/peroxisomal fractions [5], or postmitochondrial supernatants [24]. As a way of further characterizing brain catalase, we chose to relate this to our prior work with synaptosomes [16] that are isolated initially in the mitochondrial fraction from rat brain [17]. Overall, the activity of catalase was rich in the crude synaptosomal fraction with levels of activity similar to what we found in the post-mitochondrial fraction and comparable to what has been reported by others despite the range of methods used [19, 20, 23, 24]. Based on Zimatkin et al. [5], it is likely that microperoxisomes distribute between the mitochondrial and post-mitochondrial fractions in the Gray and Whittaker [17] scheme used, and our distribution of catalase activity supports that assumption. However, we were interested in whether catalase could be detected in enriched synaptosomal fractions, and indeed the activity was 2-3-fold higher in Ficoll-enriched synaptosomes compared to the crude fraction. Although enriched, it is not the level of enrichment that would indicate that all activity in the crude fraction could be accounted for by the synaptosomal components, and calculations based on yield indicated that less than 10% of the catalase in the fraction was present in synaptosomes. While the other 90% would presumably be microperoxisomes liberated from neural or glial origins (Arnold and Holtzman, 1978) during homogenization, the presence of catalase in the nerve-ending fraction is most likely due to the presence of microperoxisomes within at least some fraction of the nerve endings. Arnold and Holtzman [22] reported “catalase-positive bodies” in synaptic terminals up to P21 but indicated that they were seen more rarely in tissue from older animals. Our direct biochemical measures of catalase activity present in nerve endings would seem to support the conclusion from these early histochemical studies. We did not detect a difference between periadolescent and adult rats.

 Second, the present study is relevant to interpreting differences between adolescent and adult rats in terms of acute (intoxication related) effects of ethanol. A rather extensive series of studies have established differences in how adolescent and adult rats respond acutely to alcohol (reviewed in [13, 14]). Given that acetaldehyde is produced whenever the brain is exposed to ethanol and that it is difficult to separate some of the effects of ethanol from potential effects of acetaldehyde [1–4], one could ask whether differences in levels of brain catalase, leading to differences in acetaldehyde production or ethanol elimination, could be contributing to the observed differences between adolescents and adults in acute responses to ethanol. The present study indicates that this is unlikely because we detected no difference in baseline (control) catalase activity between adolescent rats and the corresponding adults for two commonly used rat strains.

 Third, and of direct interest for studies of chronic alcohol consumption by adolescent rats, there was no shift in catalase activity in either of the two strains when alcohol consumption began before and continued into the normal adolescent period. Work with SD rats showed that chronic alcohol consumption suppressed a number of indicators of sexual maturation [27] so we refer to this treatment group as adolescents based solely on their age at the time of testing. Catalase also remained constant in this fraction for rats that began alcohol consumption as adults. Catalase was reported to decrease after longer exposure to ethanol when measured in homogenates [23] and post-mitochondrial supernatants [24] from Wistar rats, and so we spot checked the comparable fraction from our scheme. As with the crude synaptosomal fraction, we saw no significant change in catalase activity in our post-mitochondrial fraction. Preliminary studies with Wistar rats have yielded similar findings to those reported here for the LD and SD strains (Doherty, Fathalla and Rhoads, unpublished results). We cannot rule out changes after longer exposure, but changes clearly did not occur in either fraction within the three-week administration period relevant to the appearance of strong withdrawal symptoms in the adolescent LE rats [15]. It is also of interest that we did not see shifts in catalase after either of the two very different levels of alcohol consumption seen for adolescents and adults. On average, adolescent alcohol consumption was nearly double that of the corresponding adults. This is in agreement with our earlier studies using the ethanol-containing liquid diet [15, 16]. With this liquid diet as the only source of calories, higher alcohol consumption by these adolescents may be a simple outfall of higher caloric intake associated with a period of normally high rate of growth. However, studies have shown that SD adolescents consumed double the amount of ethanol as adults in a 2-bottle choice, free-access situation [28, 29]. This is a result that cannot be ascribed to simple adolescent hyperphagia/hyperdipsia and that may be related to the differential effects of ethanol itself in adolescent and adult rats. Thus, higher levels of ethanol consumption may be the consequence of decreased sensitivity of the adolescents to effects of ethanol that might otherwise limit alcohol consumption [13]. At high levels of ethanol intake and elevated BEC, it is likely that acetaldehyde is being produced by brain catalase [5, 6]. The potential role of this acetaldehyde in the behavioral responses associated with *in vivo* ethanol administration is not addressed by the present study. However, in evaluating adaptive responses to chronic ethanol, we can now conclude that catalase is not playing a differential role in the two strains of adolescents. Moreover, the lack of effect of chronic alcohol consumption on brain synaptosomal catalase suggests that, at least in the time frame studied, regulation of brain catalase may be tuned to its physiologically “normal” role in removing H_2_O_2_ produced as a consequence of aerobic metabolism (for review see [30]) rather than what can be regarded as its “abnormal” role in ethanol metabolism. Other of the antioxidant enzymes such as superoxide dismutase and glutathione peroxidase may be more responsive to ethanol *in vivo* [23–26], and these enzymes were not tested in the present study.

## Figures and Tables

**Table 1:** Catalase activity in brain synaptosomal fractions of ethanol-naive rats: comparison of two strains and two developmental stages.

	Catalase activity (*μ*mol H_2_O_2_ min^−1^·mg protein^−1^)	
	Long-Evans	Sprague-Dawley
Preadolescent	1.42 ± 0.13	1.36 ± 0.24
Adult	1.19 ± 0.10	1.52 ± 0.16

Catalase activity (*μ*mol H_2_O_2_ min^−1^·mg protein^−1^) was determined in rat brain crude synaptosomal/mitochondrial fractions from ethanol-naïve rats and expressed as mean ± SEM. For each strain of rat, brains were isolated from two developmental stages preadolescent rats were postnatal age 25–28 days (P25–28), and adult rats were P75 or greater. There were no significant differences between strains or developmental stages.
